# ABO-incompatible kidney transplantation as a renal replacement therapy—A single low-volume center experience in Japan

**DOI:** 10.1371/journal.pone.0208638

**Published:** 2018-12-31

**Authors:** Akihiro Kosoku, Junji Uchida, Shunji Nishide, Kazuya Kabei, Hisao Shimada, Tomoaki Iwai, Nobuyuki Kuwabara, Keiko Maeda, Toshihide Naganuma, Norihiko Kumada, Yoshiaki Takemoto, Takuma Ishihara, Ayumi Shintani, Tatsuya Nakatani

**Affiliations:** 1 Department of Urology, Osaka City University Graduate School of Medicine, Osaka, Japan; 2 Department of Nursing, Osaka City University Hospital, Osaka, Japan; 3 Department of Urology, Suita Municipal Hospital, Suita, Japan; 4 Innovative and Clinical Research Promotion Center, Gifu University Hospital, Gifu, Japan; 5 Department of Medical Statistics, Osaka City University Graduate School of Medicine, Osaka, Japan; Medical University of Gdansk, POLAND

## Abstract

**Introduction:**

Living donor kidney transplantation is preferable to deceased donor transplantation due to its superior long-term patient and graft survivals. However, ABO blood group incompatibility is a major barrier to living donor kidney transplantation. ABO-incompatible kidney transplantation has been performed in Japan since the late 1980’s, but it is still globally uncommon. The objective of this study is to compare the clinical outcomes of ABO-incompatible kidney transplantation (ABO-IKT) with that of ABO-compatible kidney transplantation (ABO-CKT) at an institution where only about two kidney transplants are performed a month on average.

**Design:**

A single center propensity score-matched cohort study.

**Patients and methods:**

We retrospectively collected and analyzed the data of 240 patients with end-stage kidney disease (ESKD) who underwent living donor kidney transplantation at Osaka City University Hospital from January 1999 to December 2016, of which 66 patients were ABO-IKT. The remaining 174 patients who underwent ABO-CKT were studied as the control group, and the clinical outcomes of ABO-IKT and ABO-CKT recipients were compared based on propensity score matching.

**Results:**

After propensity score matching, there were no significant differences in both patient survival and death-censored graft survival rates between the ABO-IKT and ABO-CKT groups. Moreover, there were no significant differences in estimated glomerular filtration rate as well as frequency of acute cellular rejection, antibody-mediated rejection, infectious adverse events, malignancies, and post-operative bleeding between the two groups.

**Conclusion:**

Currently, ABO-IKT may be an acceptable treatment for patients with ESKD even at a low-volume transplant center.

## Introduction

For patients with end-stage kidney disease (ESKD), kidney transplantation is the optimal renal replacement therapy, enabling greater longevity and better quality of life compared to dialysis therapy [1–2]. The rate of living donor kidney transplants has steadily increased worldwide due to the severe shortage of deceased donors, and it is preferable to deceased donor transplantation for its superior long-term patient and graft survivals [3]. However, ABO blood group incompatibility has been a major obstacle in its implementation, and in recent years, kidney paired donation (KPD) and desensitization for ABO antibodies are being employed to overcome these immunologic barriers. Although KPD programs have become available in the United States and other countries despite ethical concerns and other issues, paired donor exchange is not allowed in Japan due to ethical reasons.

ABO-incompatible kidney transplantation (ABO-IKT) has been performed in Japan since the late 1980’s [4–5], but it is still globally uncommon. ABO-IKT is immunologically a high-risk procedure, and incidences of antibody-mediated rejection due to anti-A/B antibodies have been reported [6–7]. The immunosuppressive regimens for ABO-IKT include rituximab administration to inhibit antibody production, plasmapheresis to remove anti-A/B antibodies, and pharmacotherapy.

In this study, we compared the clinical outcomes of ABO-IKT to those of ABO-compatible living kidney transplantation (ABO-CKT) at our institution where we perform two kidney transplants a month on average.

## Patients and methods

**Fig 1:**
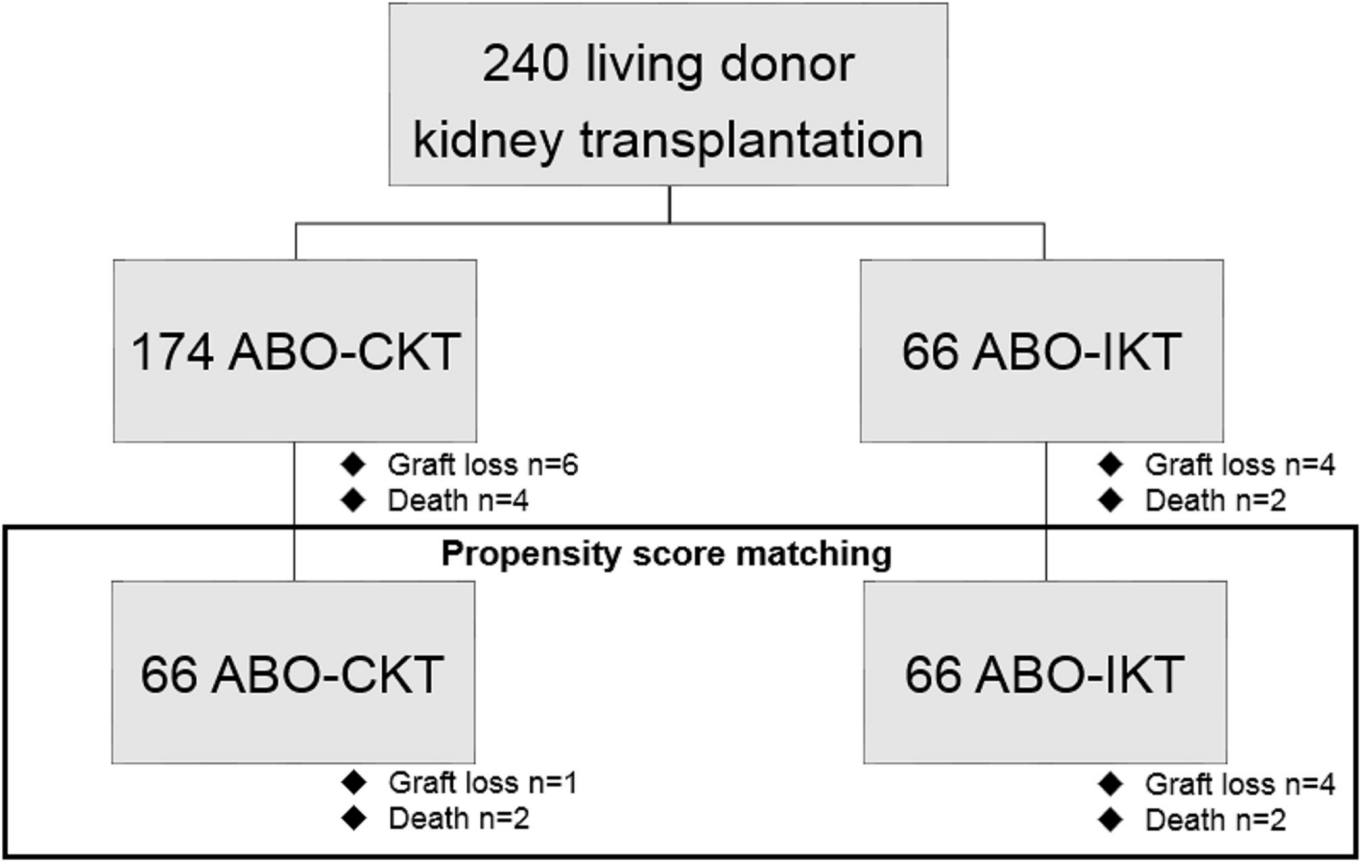
Defining the study population. Propensity score matching generated a matched cohort composed of 132 patients, 66 in each group. ABO-CKT; ABO-compatible kidney transplantation, ABO-IKT; ABO-incompatible kidney transplantation.

A total of 240 patients with ESKD underwent living donor kidney transplantation at Osaka City University Hospital from January 1999 to December 2016, of which 66 patients were ABO-IKT. The remaining 174 patients who underwent ABO-CKT were studied as the control group. It is known that quantitative and possibly qualitative differences exist in the expression of blood group A antigen in erythrocytes of A1 and A2. Because the A2 phenotype is known to be extremely rare in the Japanese population [8], we did not investigate whether they were A1 or non-A1 donor kidneys. Observation of the patients started when they underwent kidney transplantation and ended when they died or had graft loss or when the final assessments were performed on April 1st, 2017 (Fig 1).

This study was approved by the Ethics Committee of Osaka City University Graduate School of Medicine (No. 3957). Opt-out consent was obtained instead of written informed consent, which was also approved by the Ethics Committee of Osaka City University Graduate School of Medicine. We provided patients with information explaining the proposed research plan (the purpose, required individual data, and duration of research) by means of an information website of our hospital and gave them the opportunity of opt-out, and all the procedures were in accordance with the Helsinki Declaration of 2000 and the Declaration of Istanbul 2008.

### Endpoints

The primary endpoint was graft survival, censored for patient death with a functioning graft, and patient survival. The secondary endpoint was frequency of acute cellular rejection, antibody-mediated rejection, infectious adverse events, malignant tumor, and other adverse events as well as current renal function. Delayed graft function was defined as the need for at least one dialysis session within the first week of kidney transplantation. Current renal function was defined as mean estimated glomerular filtration rate (eGFR) of three most recent outpatient measurements. eGFR was calculated using the modified Modification of Diet in Renal Disease equation [9].

### ABO-CKT immunosuppression

Immunosuppressive agents have been improved since 1999, and we have used the optimal protocol available at the time. Our basic immunosuppressive protocol for ABO-CKT and ABO-IKT has consisted of three agents: calcineurin inhibitor (CNI) (tacrolimus or cyclosporine), antimetabolite (azathioprine, mycophenolate mofetil (MMF), mizoribine) or mammalian target of rapamycin (mTOR) inhibitor (everolimus (EVR)), and methylprednisolone (MP). Basiliximab (BAS) has been administered in all recipients since 2002. Briefly, CNI and MMF or EVR was initiated 3 days before transplantation. BAS was administered at a dose of 20 mg/day at the time of transplantation and 4 days after transplantation. MP was administered at a dose of 500 mg intravenously at the time of transplantation and at 40 mg/day orally 1 to 7 days after transplantation, the dose of which was decreased to 24, 12, 8, and 4 mg/day every week.

### ABO-IKT immunosuppression (Fig 2)

**Fig 2:**
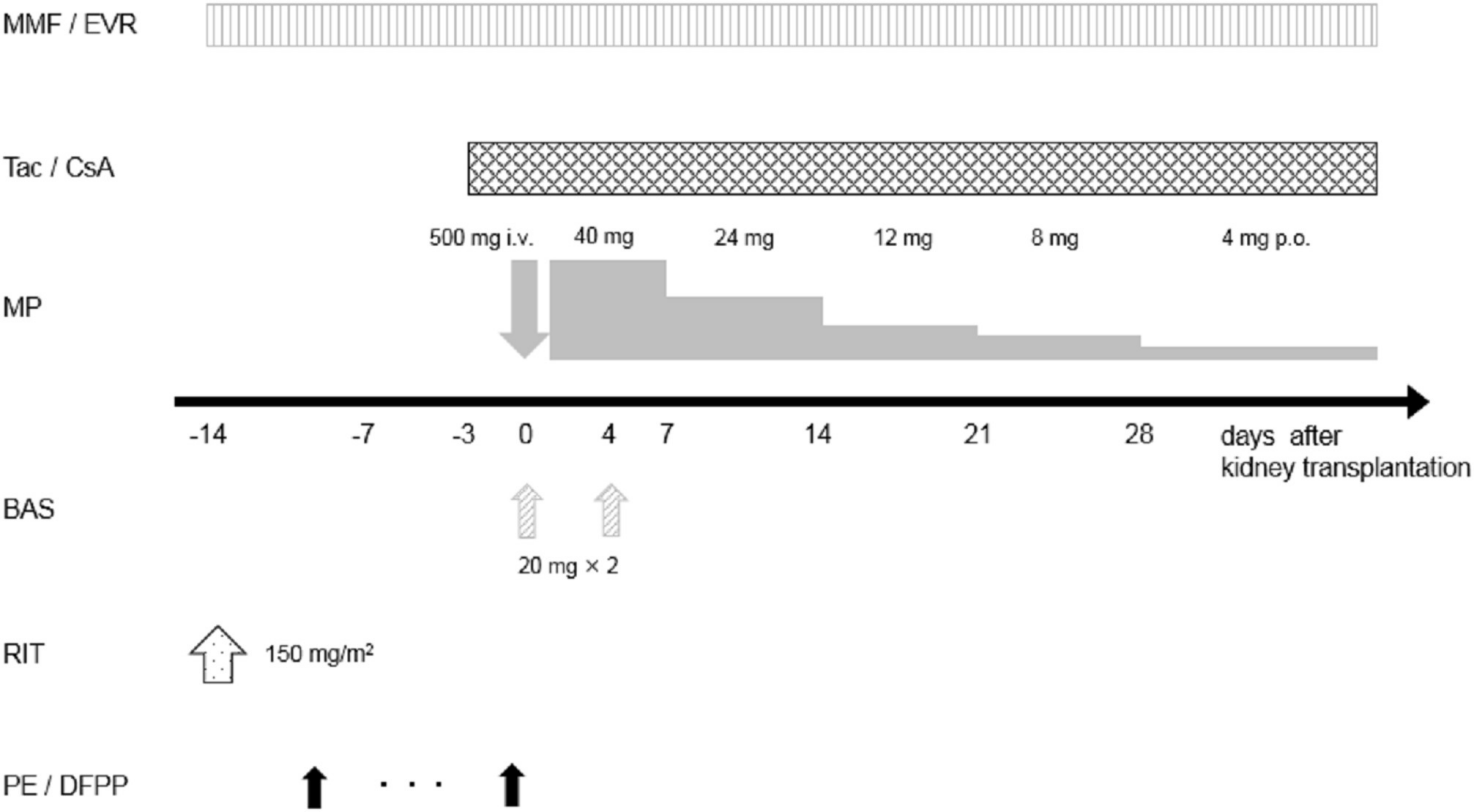
Recent basic immunosuppressive protocol for ABO-incompatible kidney transplantation. Tac; tacrolimus, CsA; cyclosporine, MMF; mycophenolate mofetil, EVR; everolimus, MP; methylprednisolone, BAS; basiliximab, RIT; rituximab, PP; plasmapheresis.

To remove the anti-A/B antibodies, the patients underwent several sessions of double filtration plasmapheresis or plasma exchange prior to kidney transplantation until the anti-A/B titers were ≤ 1:64. For the depletion of B cells and inhibition of antibody production, splenectomy was performed at the time of kidney transplantation from 1999 to 2006. Since June 2006, rituximab (anti-CD20 monoclonal antibody) has been administered instead of surgical splenectomy [10–12].

The basic protocol for ABO-IKT we currently use is shown in Fig 2. Before transplantation, antimetabolite or mTOR inhibitor was administered for 2 weeks to suppress B cell lymphocytes. Rituximab was administered at a dose of 150 mg/m^2^ 2 weeks prior to transplantation, and this basic protocol was modified depending on age and anti-A/B antibody titers. In elderly recipients (≧60 years), MMF was administered at a dose of 0.5 g/day to minimize its adverse events [13]. Some recipients with high-titers (≥ 1:512), rebound of antibody titers, or donor-specific antibodies received both splenectomy and administration of rituximab [14–15], and MMF or EVR was initiated 4 weeks before transplantation.

### Anti-A/B antibody titers of ABO-IKT

Initial (prior to desensitization), pre-operative (post-desensitization), and post-operative anti-A/B antibody titers were measured. Anti-A/B IgM antibody titers were measured using the saline agglutination test, and anti-A/B IgG antibody titers were measured using the indirect Coombs’ test.

### Biopsies and rejections

Routine biopsies of renal allografts were performed at transplantation and before the first discharge, and episode biopsies of renal allografts were performed in case of graft dysfunction. All samples were examined using light microscopy, and the samples obtained after 2006 were evaluated for deposit of C4d in the peritubular capillaries using immunofluorescent staining. Acute cellular rejection and antibody-mediated rejection were histologically diagnosed by graft biopsy specimens according to the Banff 2007–2013 criteria. Borderline changes were excluded as acute cellular rejections.

### Statistical analysis

Categorical variables were expressed as count and percentage, and continuous variables were expressed as median and interquartile range (IQR) or range. Categorical variables were compared using the Fisher’s exact test, and continuous variables were compared using the Mann-Whitney U-test. To reduce the effect of selection bias and potential confounding, propensity score matching generated a matched cohort at a 1 to 1 ratio composed of 132 patients, adjusted for recipient age at kidney transplantation, dialysis vintage, number of human leukocyte antigen mismatches, flow cytometry crossmatch positive, calcineurin inhibitor, and antimetabolite or mTOR inhibitor (Fig 1).

Survival curves were estimated using the Kaplan-Meier method, and the differences in survival rates between the two groups were evaluated using the Log-rank test. In addition, the impact of desensitization for outcomes was assessed by calculating hazard ratios and 95% confidence intervals using the univariate Cox proportional hazards regression model. P<0.05 was considered statistically significant. All statistical analyses were performed with EZR (Saitama Medical Center, Jichi Medical University, Saitama, Japan), which is a graphical user interface for R (The R Foundation for Statistical Computing, Vienna, Austria, version 3.4.1). More precisely, it is a modified version of the R commander (version 2.4–0) designed to add statistical functions frequently used in biostatistics [16].

## Results

### Baseline characteristics

The baseline clinical characteristics are shown in Table 1. The median age of the ABO-IKT and ABO-CKT recipients was 52.0 (IQR 39.3–62.0) and 44.0 (IQR 35.0–55.0) years, respectively. Graft donation from related and unrelated donors accounted for 37.9% and 62.1%, respectively, in the ABO-IKT group and 58.0% and 42.0%, respectively, in the ABO-CKT group. The age at transplant was significantly higher in the ABO-IKT recipients compared to the ABO-CKT recipients, and the percentage of graft donation from related donors was lower in the ABO-IKT recipients compared to the ABO-CKT recipients. There were no significant differences in the other clinical parameters between the two groups. The median observation period was 6.04 (IQR 2.90–10.19) years. After propensity score matching, all parameters were comparable between the two groups (Table 1), and there were no significant differences except for the factors about desensitization.

**Table 1:** Patient characteristics before and after propensity score matching.

Parameters	Before propensity score matching		p-value	After propensity score matching		p-value
	ABO-CKT(n = 174)	ABO-IKT(n = 66)		ABO-CKT(n = 66)	ABO-IKT(n = 66)	
**Recipient age at transplantation, years**	44 (IQR 35–55)	52 (IQR 39.25–62)	0.015	50 (IQR 39.25–60)	52 (IQR 39.25–62)	0.859
Recipient sex			0.883			0.471
Male	105 (60.3%)	39 (59.1%)		44 (66.7%)	39 (59.1%)	
Female	69 (39.7%)	27 (40.9%)		22 (33.3%)	27 (40.9%)	
Recipient ABO blood type			<0.001			<0.001
A	63 (36.2%)	25 (37.9%)		22 (33.3%)	25 (37.9%)	
B	48 (27.6%)	18 (27.3%)		22 (33.3%)	18 (27.3%)	
AB	29 (16.7%)	0 (0.0%)		12 (18.6%)	0 (0.0%)	
O	34 (19.5%)	23 (34.8%)		10 (15.2%)	23 (34.8%)	
**Dialysis vintage, months**	17 (IQR 5–59)	22.5 (IQR 9–53)	0.579	16.5 (IQR 7–56.25)	22.5 (IQR 9–53)	0.507
**HLA mismatch number**	3 (IQR 3–4)	4 (IQR 3–5)	0.041	4 (IQR 3–5)	4 (IQR 3–5)	0.754
**Flow cytometry crossmatch positive**	4 (2.3%)	3 (4.5%)	0.397	3 (4.5%)	3 (4.5%)	1.000
Recipient-donor relations			0.006			0.859
Related	101 (58.0%)	25 (37.9%)		27 (40.9%)	25 (37.9%)	
Unrelated	73 (42.0%)	41 (62.1%)		39 (59.1%)	40 (62.1%)	
Donor age at transplantation, years	57.5 (IQR 48.25–65)	57 (IQR 50–63.75)	0.863	57.5 (IQR 47–64)	57 (IQR 50–63.75)	0.938
Donor sex			0.764			1.000
Male	62 (35.6%)	22 (33.3%)		23 (34.8%)	22 (33.3%)	
Female	112 (64.4%)	44 (66.7%)		43 (65.2%)	44 (66.7%)	
Donor ABO blood type			<0.001			<0.001
A	52 (30.1%)	27 (40.9%)		19 (28.8%)	27 (40.9%)	
B	37 (21.4%)	20 (30.3%)		10 (15.2%)	20 (30.3%)	
AB	4 (2.3%)	19 (28.8%)		0 (0.0%)	19 (28.8%)	
O	80 (46.2%)	0 (0.0%)		37 (56.1%)	0 (0.0%)	
**Calcineurin inhibitor**			0.886			1.000
Cyclosporin	93 (53.4%)	36 (54.5%)		36 (54.5%)	36 (54.5%)	
Tacrolimus	81 (46.6%)	30 (45.5%)		30 (45.5%)	30 (45.5%)	
**Antimetabolites or Everolimus**			0.256			1.000
Azathioprine	6 (3.4%)	1 (1.5%)		1 (1.5%)	1 (1.5%)	
Mycophenolate mofetil	147 (84.5%)	62 (93.9%)		61 (92.4%)	62 (93.9%)	
Mizoribine	7 (4.0%)	0 (0.0%)		0 (0.0%)	0 (0.0%)	
Everolimus	14 (8.0%)	3 (4.5%)		4 (6.1%)	3 (4.5%)	
Basiliximab	158 (90.8%)	63 (95.5%)	0.293	65 (98.5%)	63 (95.5%)	0.619

ABO-CKT; ABO-compatible kidney transplantation, ABO-IKT; ABO-incompatible kidney transplantation, HLA; human leukocyte antigen.

### Patient and graft survivals

**Fig 3:**
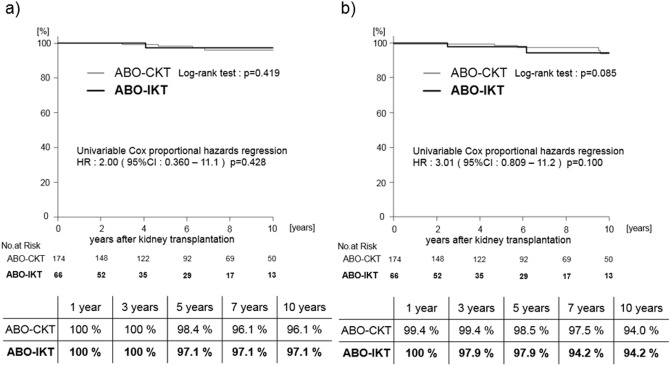
Patient and graft survival rates of ABO-CKT and ABO-IKT before propensity score matching. (a) Patient survival rates (b) Graft survival rates. ABO-CKT; ABO-compatible kidney transplantation, ABO-IKT; ABO-incompatible kidney transplantation.

In the ABO-CKT group, 4 patients died (subarachnoid hemorrhage, heart failure, acute pancreatitis due to alcohol, peritonitis), and in the ABO-IKT group, 2 patients died (myelodysplastic syndrome, heart failure). Patient survival rates at 1, 3, 5, 7, and 10 years were 100%, 100%, 98.4%, 96.1%, and 96.1%, respectively, in the ABO-CKT group and 100%, 100%, 97.1%, 97.1%, and 97.1%, respectively, in the ABO-IKT group, and there was no significant difference between the two groups (Fig 3(a)). Four and 6 patients lost graft function excluding death with graft function and were reintroduced to dialysis in the ABO-CKT group and ABO-IKT group, respectively. The death-censored graft survival rates at 1, 3, 5, 7, and 10 years were 99.4%, 99.4%, 98.5%, 97.5%, and 94.0%, respectively, in the ABO-CKT group and 100%, 97.9%, 97.9%, 94.2%, and 94.2%, respectively, in the ABO-IKT group, and there was no significant difference between the two groups (Fig 3(b)).

**Fig 4:**
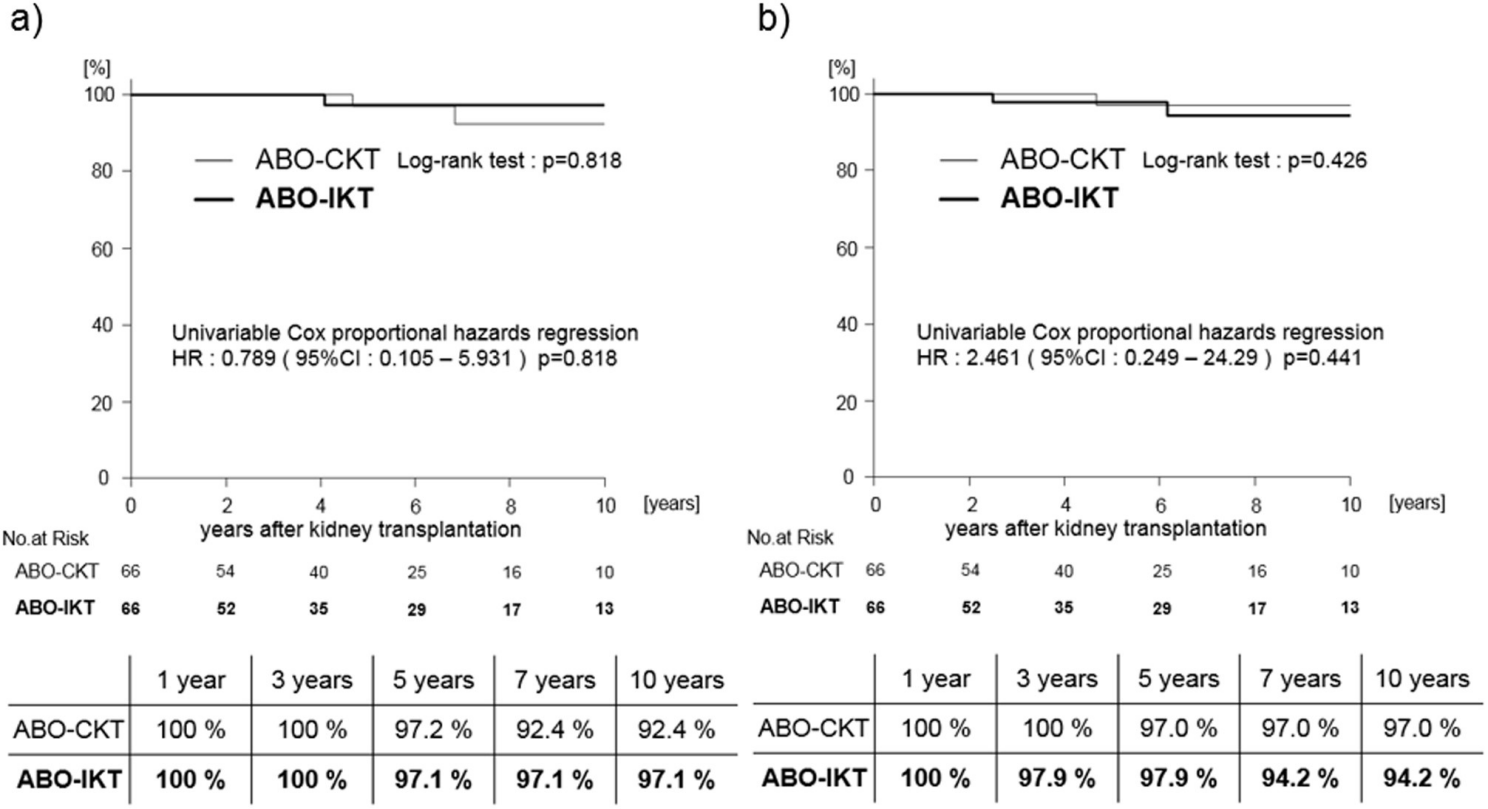
Patient and graft survival rates of ABO-CKT and ABO-IKT after propensity score matching. (a) Patient survival rates (b) Graft survival rates. ABO-CKT; ABO-compatible kidney transplantation, ABO-IKT; ABO-incompatible kidney transplantation.

After propensity score matching, the patient and graft survival rates were compared. The median observation period was 4.54 (IQR 2.40–7.44) years in the matched cohort. During the period of follow-up, 1 and 4 recipients were reintroduced to dialysis and the number of deaths with a functioning graft was 2 and 2 in the ABO-IKT group and ABO-CKT group, respectively. Patient survival rates at 1, 3, 5, 7, and 10 years were 100%, 100%, 97.0%, 92.4%, and 92.4%, respectively, in the ABO-CKT group and 100%, 100%, 97.1%, 97.1%, and 97.1%, respectively, in the ABO-IKT group, and there was no significant difference between the two groups (Fig 4(a)). The death-censored graft survival rates at 1, 3, 5, 7, and 10 years were 100%, 100%, 97.0%, 97.0%, and 97.0%, respectively, in the ABO-CKT group and 100%, 97.9%, 97.9%, 94.2%, and 94.2%, respectively, in the ABO-IKT group, and there was no significant difference between the two groups (Fig 4(b)).

### Renal function and rejection episodes (Table 2)

**Table 2:** Comparison of renal function and rejection episodes between ABO-CKT and ABO-IKT after propensity score matching.

	ABO-CKT (n = 66)	ABO-IKT (n = 66)	p-value
Acute cellular rejection	7 (10.6%)	14 (21.2%)	0.152
Steroid-resistant acute cellular rejection	3 (4.5%)	6 (9.1%)	0.492
Acute antibody-mediated rejection	0 (0.0%)	2 (3.0%)	0.496
Renal function (estimated glomerular filtration rate), mL/min/1.73m^2^			
1 year after transplantation	47.61 (IQR 39.25–57.45)	45.03 (IQR 38.23–54.14)	0.230
3 years after transplantation	48.20 (IQR 41.22–59.73)	46.90 (IQR 40.95–54.50)	0.348
5 years after transplantation	50.69 (IQR 44.54–59.53)	48.47 (IQR 41.90–51.45)	0.181
7 years after transplantation	53.56 (IQR 48.88–59.70)	48.64 (IQR 43.14–54.54)	0.099
10 years after transplantation	53.89 (IQR 50.17–59.09)	49.24 (IQR 40.78–52.20)	0.094
Current	45.65 (IQR 37.61–57.61)	44.06 (IQR 37.86–51.35)	0.363

ABO-CKT; ABO-compatible kidney transplantation, ABO-IKT; ABO-incompatible kidney transplantation.

There were no significant differences in eGFR at 1, 3, 5, 7, and 10 years after transplantation between the ABO-CKT and ABO-IKT groups after propensity score matching. Current renal function (eGFR) of the ABO-CKT and ABO-IKT recipients was 45.65 (IQR 37.61–57.61) and 44.06 (IQR 37.86–51.35) mL/min/1.73 m^2^ after propensity score matching, respectively, and the ABO-IKT group did not significantly differ from the ABO-CKT group (p = 0.363).

The rate of acute cellular rejection in the ABO-CKT and ABO-IKT groups was 10.6% and 21.2%, respectively, after propensity score matching, and there was no significant difference between the two groups. Four recipients in the ABO-CKT group and 6 in the ABO-IKT group developed steroid-resistant acute cellular rejection requiring OKT-3 or anti-human thymocyte immunoglobulin. Although the incidence of steroid-resistant rejection in the ABO-IKT group was higher than that in the ABO-CKT group (p = 0.0285, 2.5% vs 9.1%, ABO-CKT group vs ABO-IKT group) before propensity score matching, there was no significant change in the rate of steroid-resistance acute rejection between the two groups after propensity score matching.

Two recipients experienced antibody-mediated rejection due to anti-A/B antibody titers. They developed antibody-mediated rejection without an explosive post-operative elevation in anti-A/B antibody titer (1:16 to 1:64 and 1:8 to 1:32, respectively) and were successfully treated with steroid pulse therapy, plasma exchange, intravenous administration of immunoglobulin, and/or rituximab administration. The incidence of antibody-mediated rejection in the ABO-CKT and ABO-IKT groups was 0% and 3.0% after propensity score matching, respectively. Accordingly, there were no significant differences in the rates of antibody-mediated rejection between the two groups.

### Desensitization protocol and changes of anti-A/B antibody titers in ABO-IKT recipients (Table 3)

**Table 3:** Desensitization for ABO-IKT recipients.

			ABO-IKT (n = 66)
B cell depletion	Splenectomy		8 (12.1%)
	Rituximab		48 (72.7%)
	Rituximab + Splenectomy		10 (15.2%)
Sessions of plasmapheresis			4 (range 1–13)
Anti-A/B antibody titers	Initial antibody titers	IgG	32 (range 1–4096)
		IgM	16 (range 2–512)
	Preoperative antibody titers	IgG	2 (range <1–64)
		IgM	1 (range <1–8)

ABO-IKT; ABO-incompatible kidney transplantation

Of the 66 ABO-IKT recipients, 8 patients received splenectomy, 48 received administration of rituximab, and 10 received both splenectomy and administration of rituximab for B cell depletion. They also underwent 1 to 13 sessions of plasmapheresis to remove the anti-A/B antibodies. The range of initial anti-IgM and IgG A/B antibody titers was from 1:1 to 1:4096 and from 1:2 to 1:512, respectively, and our desensitization protocol achieved pre-operative A/B antibody titers of less than 1:64. Except for the two recipients who underwent antibody-mediated rejection, all the recipients experienced no significant post-operative anti-A/B antibody titer elevation causing antibody-mediated rejection.

### Complications (Table 4)

**Table 4:** Comparison of complications between ABO-CKT and ABO-IKT after propensity score matching.

	ABO-CKT (n = 66)	ABO-IKT (n = 66)	p-value
Adenovirus urinary tract infection	3 (4.5%)	6 (9.1%)	0.492
Pneumocystis pneumonia	2 (3.0%)	4 (6.1%)	0.680
BK virus nephropathy	0 (0.0%)	0 (0.0%)	—
Cytomegalovirus infection	35 (53.0%)	41 (62.1%)	0.379
Cytomegalovirus disease	0 (0.0%)	2 (3.0%)	0.496
Malignant tumor	5 (7.6%)	3 (4.5%)	0.718
Delayed graft function	2 (3.0%)	3 (4.5%)	1.000
Surgical treatment for postoperative hemorrhage	2 (3.0%)	4 (6.1%)	0.680

ABO-CKT; ABO-compatible kidney transplantation, ABO-IKT; ABO-incompatible kidney transplantation

Cytomegalovirus (CMV) infection occurred in 50.0% of the ABO-CKT recipients and 62.0% (p = 0.219) of the ABO-IKT recipients. Two patients who received splenectomy as desensitization underwent very late-onset CMV disease: one experienced CMV colitis 15 years after transplantation, and one had CMV retinitis 9 years and 9 months after transplantation. There were no significant differences in the rates of adenovirus urinary tract infection, pneumocystis pneumonia, BK virus nephropathy, malignancies, delayed graft function, and post-operative bleeding between the two groups.

## Discussion

In this present study, we compared the clinical outcomes between ABO-IKT and ABO-CKT recipients based on propensity score matching. As a result, there were no significant differences in graft and patient survival rates between the two groups. These results suggested that ABO-IKT may be an acceptable treatment for patients with ESKD even at an institution where only about two kidney transplants a month are performed on average.

Due to the short supply of deceased donor grafts, living donor kidney transplantation accounts for about 30% of all kidney transplants performed in the United States and for approximately 90% in Japan [17–18]. The outcomes of living donor kidney transplantation are significantly better than those of deceased donor transplantation [3], but unfortunately, many living donor/recipient pair transplants are unacceptable because of ABO-incompatibility and pre-existing donor specific antibodies. Two strategies to overcome these barriers are KPD and desensitization, which can be used individually or in combination.

There have been concerns about the suboptimal long-term outcomes of ABO-IKT which requires desensitization. Opelz et al reported that early patient survival was lower in ABO-IKT than in ABO-CKT due to a higher rate of early infectious deaths, although patient and death-censored graft survival rates were similar, by analyzing the international Collaborative Transplant Study (CTS) database [19]. In a U.S. national cohort study, ABO-IKT and ABO-CKT outcomes were not significantly different, but the risk of death-censored graft survival was higher in ABO-IKT [20]. Our study demonstrated that patient survival rates at 3 years were 100% in both the ABO-CKT and ABO-IKT groups, and there were no significant differences in early patient survival rates before and after propensity score matching between the two groups. Moreover, we revealed that the death-censored graft survival rate at 10 years was 97.0% in the ABO-CKT group and 94.2% in the ABO-IKT group, and there was no significant difference between the two groups. In a Japanese national cohort study, long-term patient and graft survivals were not significantly different between the ABO-CKT and ABO-IKT groups [21]. Okumi et al. showed that long-term graft survival of ABO-IKT recipients was almost identical to that of ABO-CKT recipients over the past decade due to evolution of outcomes and immunosuppressive management in a high-volume single center study [22]. Appropriate desensitization for ABO-IKT may not be a disadvantage for patient and graft survival rates, although a follow-up of over 10 years is necessary to confirm this matter.

Desensitization protocols for ABO-IKT had not been established until recently. Our desensitization protocol consists of immune-modulation to remove anti-A/B antibodies prior to transplantation, B cell depletion therapy (splenectomy and/or rituximab administration), and pharmacotherapy. ABO-IKT is immunologically a high-risk procedure because of ABO incompatibility, but intensive immunosuppressive protocols for ABO-IKT may cause over-immunosuppression. We therefore modify our basic protocol depending on recipient age, rebound of antibody titers, or baseline antibody titer [13–15]. Although it may be difficult to establish a definitive immunosuppressive therapy for ABO-IKT, we should develop a tailored desensitization protocol for preventing infections and avoiding rejections in these recipients with various conditions. In fact, everolimus may be a useful immunosuppressant for ABO-IKT [23].

In our study, only two patients underwent antibody-mediated rejection by anti-A/B antibodies and were successfully treated with intensive treatment including plasmapheresis. We did not experience graft loss due to antibody-mediated rejection with an explosive elevation in anti-A/B antibody titer. These results suggested that our intensive desensitization for ABO-IKT including B cell depletion, plasmapheresis, and pharmacotherapy may prevent intractable antibody-mediated rejection. On the other hand, steroid-resistant acute cellular rejection requiring OKT-3 or anti-human thymocyte immunoglobulin was more frequent in the ABO-IKT group than in the ABO-CKT group in the present study before propensity score matching. However, there was no significant difference in steroid-resistant acute cellular rejection between the two groups after propensity score matching (Table 2). The incidence of rejection episodes in the ABO-IKT recipients may be comparable to that of the ABO-CKT recipients because of the recent evolution in immunosuppressive therapies.

There was no significant difference in the rate of infectious complications including CMV between the two groups in our study, although intensified desensitization for ABO-IKT has been reported to be associated with an increased risk of infectious complications [24–26]. Splenectomy was performed at the time of kidney transplantation from 1999 to 2006 at our institution, and two patients who underwent splenectomy developed very late-onset CMV disease, which may have been caused by this procedure. Since June 2006, we have administered rituximab instead of performing splenectomy. Previous reports also showed a significant increase in the rate of infectious complications such as CMV among kidney transplant recipients receiving rituximab [27]. However, in randomized, double-blind, placebo-controlled studies, ABO-CKT recipients randomized to receive one dose of rituximab had no increase in the incidence of infections and malignancies [28–29]. It is possible that we did not detect a significant difference in the rate of infectious complications between ABO-IKT and ABO-CKT due to the small size of our study. In any case, we found no critical infectious complications in our ABO-IKT recipients during our observation period (median 6.04 years).

Recently, there have been various studies to avoid excessive immunosuppression due to desensitization, not only without B cell depletion therapy [30–31], but also without removal of anti-A/B antibodies [32]. Masterson R et al. reported that patients with low anti-A/B antibody titers could undergo ABO-IKT with standard immunosuppression alone [32]. Sautenet B et al. showed no benefit of rituximab on treatment of antibody mediated rejection [33]. It might therefore become possible to simplify desensitization protocols in the future. However, patients with low anti-A/B antibody titers undergoing ABO-IKT without desensitization had acute antibody-mediated rejection, resulting in graft loss [34].

KPD enables kidney transplant candidates with willing but incompatible living donors to join a registry of other incompatible pairs in order to find potentially compatible transplant donors. Single-center KPD has been reported from several countries including India, Romania, and Turkey. Multicenter or national KPD programs exist in many countries including the United States, Canada, Australia, the Netherlands, the United Kingdom, and Switzerland [35–36]. Pairs are entered into the KPD registry because of ABO-incompatibility, and a better immunological match can result in long-term graft survival and less economical strain due to less use of immunosuppressants by avoiding intensive desensitization. However, blood type O and/or highly-sensitized patients may have a disadvantage with a limited number of donors and match rates as low as 15%, resulting in a prolonged waiting time for transplantation [37]. The match rates of KPD depend on the KPD pool size and KPD pool donor-recipient composition, and typically only 50% of pairs can be matched even in larger programs. A previous report revealed that all of the registrants had been waiting for more than 1 year at a mean of 747±322 days [38]. Transplant outcomes of KPD may be worse than those of ABO-IKT due to a longer waiting time of over 6 months, but large-scale clinical trials comparing ABO-IKT and KPD are needed [39–40].

KPD has some ethical issues, because the kidney of the donor may not be transplanted to the intended recipient but to an unknown recipient. Furthermore, coercion has been a concern for living donation, and it is possible that KPD places donors under even greater pressure to donate, because incompatibility is eliminated as an excuse to avoid donation [41]. There are further ethical concerns such as privacy, confidentiality, exploitation, and commercialization [42]. In this study, we found that there were no significant differences in kidney transplant outcomes between ABO-IKT and ABO-CKT at our institution. KPD is not permitted in Japan because of ethical reasons, and it might be unnecessary to implement such a program in this country.

There may be several limitations in the present study. The study was conducted using a retrospective design, and the number of patients enrolled in this study was small. Despite these limitations, excellent clinical outcomes of ABO-IKT were obtained by propensity score matching which were not significantly different compared with those of ABO-CKT at our institution where we perform only about two kidney transplants a month on average.

In conclusion, at present, ABO-IKT may be an acceptable treatment for patients with ESKD even at a low-volume transplant center. ABO-IKT may increase the frequency of kidney transplants and may lead to the shortening of dialysis therapy or even its avoidance. Because KPD is not allowed in Japan due to ethical reasons, such a program may not be necessary, but further studies are needed to clarify this matter.
